# An Agenda for Closing Resource Gaps in Global Mental Health: Innovation, Capacity Building, and Partnerships

**DOI:** 10.3109/10673229.2012.652875

**Published:** 2012-02-15

**Authors:** Anne E Becker, Arthur Kleinman

**Affiliations:** * Department of Global Health and Social Medicine, Harvard Medical School; Department of Psychiatry, Massachusetts General Hospital, Boston, MA. Email: anne_becker@hms.harvard.edu; † Department of Global Health and Social Medicine, Harvard Medical School; Asia Center and Department of Anthropology, Harvard University. Email: kleinman@wjh.harvard.edu

The high global burden of mental disorders has been well-documented since the 1990s, with neuropsychiatric disorders presently comprising approximately 13% of the world's health burden.^1^ Four mental disorders rank among the ten leading causes of years lost due to disability in low- and middle-income countries—including the unipolar depressive disorders, which rank first.^1^

Notwithstanding this visibility of burden, the field of global mental health can also be characterized by its inordinate resource gaps resulting in tragically high and persistent unmet needs. For example, across the entire economic spectrum—and especially in low- and middle-income countries—public sector resource allocation for mental health is disproportionately low,^2^ and this from a pie that is already much too small. With comparatively meager economic resources and also the social stigma attached to mental disorders, profound shortfalls in the human resources needed for adequate mental health care in low-resource settings are unsurprising but unacceptable.^3^ The mental health treatment gap—a proxy for the mismatch of disease burden with extant resources—stands as disconcerting evidence of a major failure in global health delivery. More than three-quarters of individuals with serious cases of mental illness in less-developed countries appear not to receive treatment for those problems.^4^

Global mental health is also dogged by a research gap that, while less visible, has had tangible adverse impact. Populations outside of Europe and North America receive little coverage in high-impact psychiatry journals. In fact, only 6% of content covers approximately 90% of the world's population,^5^ making little room to advance a research agenda that would represent the mental health care priorities in low- and middle-income countries. This is a critical deficit since the treatment gap cannot be resolved by extending presently available services alone. Also essential will be the adaptation of treatments and the development of novel service-delivery models with greater local relevance and empirically supported effectiveness.

This special issue of the *Harvard Review of Psychiatry* presents a collection of seven articles by leading scholars and implementers who are engaged in meeting the enormous challenges imposed by unmet world needs in delivering mental health care. The special issue is themed around relevant and pressing resource gaps, the strategies and steps that have been set into motion to close them, and the work and commitments that are still needed. In aggregate, these articles set out a convincing argument that more than just a greater share of health resources will be necessary to erase the enormous resource deficits and inequities characterizing global mental health care. Rather, innovative approaches to mental health services are required, including interventions that encompass both clinical and social domains of action. These approaches, moreover, will require a robust empirical base supporting their local effectiveness and feasibility. Finally, in-country research and training are necessary, and clinical infrastructure and capacity must be built.

In this issue's first article, “Global Mental Health: From Science to Action,” Patel sketches out a brief history of recent milestones that have “galvanized” the field of global mental health in the domains of advocacy, treatment, and research. These milestones include the World Health Organization's Mental Health Gap Action Programme (mhGAP), rolled out in 2008 as a means of scaling up care for major mental disorders; the Movement for Global Mental Health, developed as a means of raising the visibility of, and lending voice to, exigent global mental health needs and inequities; and the focused global mental health research agenda constructed by the Grand Challenges in Global Mental Health. Patel's piece emphasizes that innovation will be essential to overcoming the remaining major barriers to meeting global mental health care needs.

In their article, “Capacity Building in Global Mental Health Research,” Thornicroft and colleagues cite the urgency and enormity of need to build in-country research capacity that can support high-quality and locally relevant mental health research at the individual, institutional, and national levels. They argue that deficits in mental health research capacity are especially steep because of the field's universal marginality. Thornicroft and colleagues outline a series of steps that are straightforward, wise, and pragmatic to address these deficits and that include skills transfer, monitoring and evaluation, and engaging the participation of both junior and senior personnel.

In “Relevance or Excellence? Setting Research Priorities for Mental Health and Psychosocial Support in Humanitarian Settings,” Tol and colleagues present findings from their study eliciting local research priorities from policymakers, investigators, and humanitarian aid workers in Nepal, Peru, and Uganda, countries that had each undergone a recent humanitarian crisis. Whereas a consensus emerged around the mental health research topics of highest concern, the study also identified an incongruence in preference for research that could have immediate utilitarian value versus investigations that might have theoretical or general importance. This study therefore highlights the imperative not only of bridging resource gaps but also of navigating competing demands for “relevance” and “excellence” in research.

The next two articles in this issue describe models for partnerships between high- and low-income countries that can build local mental health care clinical, research, and training capacities in resource-constrained regions. In “The Centre for International Mental Health Approach to Mental Health System Development,” Minas sets forth the approach developed by the center, established in 1996 and institutionally situated in the Melbourne School of Population Health. The program supports key platforms for leadership training, research capacity building, collaborative networking for mental health policy research, and disseminating empirical data on mental health systems through a journal and conferences specifically intended to benefit low-resource countries. In a case example drawn from Sri Lanka, Minas describes how these components were integrated into an effort to develop the country's mental health care system. Minas offers additional summary metrics and illustrations of the impressive output from this program.

Fricchione and colleagues' “Capacity Building in Global Mental Health: Professional Training” is both exhortation and playbook to create partnerships linking institutions in high- and low- or middle-income countries—a collaborative training model known as *educational twinning*. They outline features of low- and middle-income countries' health care systems that undermine development of a cadre of well-trained psychiatrists, and advocate a comprehensive training framework for mental health service delivery that spans the full educational spectrum in-country. They also outline the benefits of global mental health professional development in high-income countries—which can be utilized to complement and advance mental health professional training in resource-constrained regions. They illustrate a successful institutional collaboration between the Departments of Psychiatry at the University of Toronto and Addis Ababa University, which led to the establishment of Ethiopia's first psychiatry residency training program. They argue that similarly successful partnerships will require focused attention on clinical training materials and research training. Such efforts will also require broad-based participation and engagement among academic institutions in high-income countries.

The final two articles discuss programmatic implementation in two resource-constrained settings with extraordinary mental health needs: Tomsk (Russia) and Haiti. Both articles reflect upon on-the-ground challenges that set resource-constrained settings apart from well-resourced health systems, with particular emphasis on program implementation and, more generally, the key strategies that can be used to address those challenges. In “Implementing Evidence-Based Alcohol Interventions in a Resource-Limited Setting: Novel Delivery Strategies in Tomsk, Russia,” Shin and colleagues describe the well-conceived, step-by-step adaption, implementation, and evaluation of an innovative intervention for alcohol abuse integrated into an extant tuberculosis care program. In addition to discussing the structural barriers regarding access to mental health services that are especially germane for socially marginalized patients, the authors detail their painstaking efforts to render both psychosocial and pharmacologic interventions locally acceptable and effective. They conclude by reflecting on the elements of their implementation process that led to its demonstrated success, including local engagement, collaborative needs assessment, integration into the existing health care infrastructure, task shifting to nonspecialized providers, “accompaniment” throughout the implementation process, and securing a leadership commitment to sustaining the intervention.

Raviola and colleagues' “Mental Health Response in Haiti in the Aftermath of the 2010 Earthquake: A Case Study for Building Long-Term Solutions” describes a broadly coordinated response to the unprecedented mental health care needs in Haiti following its recent catastrophic earthquake. This response was organized around a locally driven agenda for clinical care, with corresponding attunement to local research priorities and to economic and human resource constraints. In this respect, this extraordinary case is an exemplar of effective integration of research, service, training, and advocacy, along with engagement of local knowledge, expertise, and collaboration, to address exigent mental health needs in the aftermath of a natural disaster and in a setting of chronic poverty.

There would be little challenge to a declaration of urgency and dire need in the field of global mental health: the burden of disease is high, and existing resources are both scarce and deficient. Those facts are plain enough, even if the means of redressing them have not been. Against that background, the articles in this issue provide especially important and timely guidance in asserting the tandem need for innovation and capacity building to develop and implement locally relevant, feasible, and effective mental health care. This innovation and capacity building must also integrate multiple domains of service delivery, health systems management, clinical training, and research. Capacity building, in turn, requires leadership, resources, and institutional commitment, as well as partnerships and serious, sustained commitments from within high-income countries, if global expertise and experience are to respond effectively to local priorities and needs.

The issues that we highlight in this special issue are necessary, but probably not sufficient, to reshape the global response to mental health problems. In addition, what is needed is understanding of those political, economic, and cultural barriers that have for so long impeded global mental health care and that have placed it at serious disadvantage within global health care writ large. Among these barriers—which, for so long, have allowed an outrageous reality to persist with so little outcry—are a politics of indifference and the ravages of stigma (emphasized by Patel in his piece), which becomes a social death to sufferers of the most serious mental illnesses. Overcoming these many challenges will require the kind of political activism for which the HIV/AIDS movement has been such a sterling example. It will also require consciousness raising—to which this special issue contributes by its very publication.
